# The Position and Future of the Panel

**Published:** 1913-09-06

**Authors:** 


### THE POSITION AND FUTURE OF THE PANEL.

### A Survey of Conditions in Town and Country.

In 1912 the representative body of the British Medical Association, with the impulsiveness due to its democratic constitution, asserted by an overwhelming majority that the Insurance Act "was unworkable and derogatory to the medical profession." In 1913 Mr. Lloyd George, with the impulsiveness due to his Celtic origin, declared that his Act was working most satisfactorily, and that he had 18,000 medical men on his panels. For the prospective student of medicine it is important that these two apparently irreconcilable statements should be investigated, and a search made for the truth as regards the present position and future prospects of the panel system in this country.

The fact that a very large number of medical men are working under the Insurance Act shows that the word "unworkable" was too strong, or at all events needs qualifying. On the other hand, when Mr. Lloyd George instances the size of his panels as a proof of the satisfactory working of the Act, he carefully suppresses the fact that very many medical men had no alternative but to put their names on the list, and to do what they could under conditions that they loathed. Has their experience of the work materially lessened this loathing? Does it suggest grounds for hope in the future? It is possible that the work of the 18,000 is no better, possibly inferior, to that performed before the existence of the Act; and that the conditions are so derogatory that it would have been better for the community in the end if the machinery for medical benefit had proved absolutely unworkable.

First of all, it may be admitted at once that the terms of remuneration under the Act have led to improvement in the income of a certain number of men for a certain class of work. In other cases the result of the changed conditions of payment has been to leave matters pretty much as they were. Only in better-class districts, where the provisions of the Act have gained very little foothold so far, is there a probability that incomes will actually be reduced if the panel system ever becomes properly established. As an instance of increased remuneration, we may take a practice in a purely industrial area, where a firm of doctors has been in the habit of seeing large numbers of friendly society subscribers in the usual kind of surgery. The patients come in their tens or hundreds at certain fixed hours; they were seen at a very rapid rate; the majority, having only small ailments, were dismissed quickly with an order for some fairly innocuous medicine; the few whose symptoms suggested more serious disease were held over, and their cases gone into a little more thoroughly. The system worked fairly well, and instances of grave disease only occasionally slipped through unexamined. It was the best treatment that could be expected for the sum of 4s. per head per annum. Under the Insurance Act the same system is being carried out on the same people, in the same buildings, under the same conditions of treatment; but the remuneration is no longer 4s. but 7s. per head. Moreover, many people who were unable to pay their bills are now insured persons, and help to swell the doctor's income; while the better-class patient, who has become involved in the meshes of the Act, declines to put up with wholesale treatment in a barn-like surgery, and consequently consents to forfeit the benefit to which he has contributed, continues to be a private patient, and pays his private fee in addition to his Insurance Act tax.

Take, again, the case of the rural labourer. He is generally a healthy person, but 14s. a week does not permit of the payment of fees when he happens to be ill. The doctor now receives 7s. a year for him. There is no alteration in treatment, no improvement of conditions; but the doctor is better paid, the labourer is soothed by receiving sick benefit, and everything goes on otherwise in the same old way. So much for the question of remuneration; and if there were nothing else in the Act but increased pay for the same kind of work as heretofore on patients who were formerly members of friendly societies or unable to pay decently remunerative fees, there would be little to be said.

### The Cloud on the Horizon.

But the doctor is not far-sighted enough to descry the cloud on the horizon. That cloud, which may appear in places small enough at present, is the cloud of Government control. The panel doctor is apt to forget that he is now a State servant, paid for certain work by the State; that the work was intended by the promoters of the Act to be better work than he has done heretofore; that the Government, his master, is represented by the labourer, the friendly society nominee, and the insurance company tout; that the Act is yet young; and that these representatives of the Government do not fully appreciate as yet their powers. The medical man fails to recognise that some day Insurance Committees, drawn from a lower class than at present, but with a fuller knowledge of the "rare and refreshing fruit" to which they are entitled, will begin to put pressure on the profession, and to demand from the panel doctor the full value of the 7s. For it is plain that under this Act a large proportion of the medical men of this country have sold themselves into an enormous extension of contract work.

The chief meaning of the Insurance Act as at present administered is that at least three-quarters of all the practitioners in this country will be made *compulsorily* to undertake contract work under the supervision and control of Committees, mainly composed of laymen, with Government authority at their back. While such a proposition has no terrors for certain political doctrinaires, there are many to whom it seems by no means unfraught with danger. It would be well to explain to those who have not an intimate knowledge of the profession of medicine the nature of the varieties of ordinary general practice. In the first place, there is the private practice which has aroused the gibes of Mr. Bernard Shaw and his like, where the patient pays for such attendance as he receives, the payments being calculated at so much per attendance. Secondly, there is the form of practice known as contract work, a method hitherto associated with friendly societies and other such bodies. This is a form of insurance primarily intended for those whose means do not permit of the payment of doctor's bills. Every member of the society or club contributes a fixed sum yearly, which is either handed over to the doctor for each prospective patient whose name is on his list; or the contributions are pooled, and any medical man attending a member of the society is paid at a fixed rate for each attendance on that member. Finally, there is the method of whole-time medical officers, who are paid an annual salary for undertaking the treatment of any of the members of the society who may happen to fall sick during the year.

The method of practice adopted under the Insurance Act is the first variety of the second type—that is, each doctor who puts his name on the panel is supposed to receive the sum of 7s. annually for every insured person who is received as a possible patient, and for the aggregate of these sums he is supposed to provide a somewhat extensive amount of treatment for everyone on his list who may happen to fall ill. This system is nothing but a vast extension of the old contract practice under the friendly societies, and the method of payment is known as the capitation system. It is over the principle of contract practice that a great deal of the warfare against the Insurance Act has taken place. The attitude of the majority of doctors may be summed up in the following syllogism: Contract practice is bad. Some measure of contract practice is inevitable. Therefore contract practice should be kept within the narrowest limits possible.

If this conclusion is accepted, what is to be said of an Act which has extended the principles of contract treatment from the 6,000,000 original members of friendly societies to 14,000,000 insured persons, while its authors propose at the earliest possible opportunity to include the dependants of these 14,000,000 persons under the same form of practice? It means that out of 41,000,000 inhabitants of this country 36,000,000 to 37,000,000 will receive treatment on contract terms; and it is understating the case to say that at least three-quarters of the medical profession will be compelled to be on the panel.

It is in the better-class districts in large cities that the chief troubles in getting the Insurance Act to work have arisen. Hence the poverty of the panel in the West End of London, Edinburgh, and certain other places. In such districts not only is the doctor unaccustomed to contract work, but the insured persons, consisting of clerks, typists, secretaries, domestic servants, and the sons and daughters of the well-to-do, have always been in the habit of paying their own fees, have been in most instances perfectly well able to do so, have been transferred to the comfort of a hospital in prolonged or serious illness, and neither from hearsay nor from observation have been able to discover any advantage in treatment on contract terms. The half million insured persons in London who have not sought for a doctor on the panel include tens of thousands of these patients. So fearful are they of contamination by the Act that they do not even apply in many cases for their sick pay. Thus a lady secretary who was laid up for a time last spring was under the impression that only a panel doctor could obtain for her the promised 7s. 6d. a week, and consequently she did not breathe a word to her non-panel doctor that she was an insured person lest he might transfer her to some one on the panel. Cases of this sort are numerous in better-class districts, and neutralise the full effects of malingering.

If, however, by any chance the panel should come to occupy the position originally designed for it, if panel treatment at 7s. a year should ever become similar to the treatment meted out to dukes, then the insured person who preferred to maintain his spirit of independence and to decline the State service offered to him would indeed be foolish. It is not yet fully recognised that if a wealthy stockbroker employs his son in his office at a salary of less than £160 a year, that son is an insured person, and as such entitled to medical attendance and drugs under the Insurance Act. With an idealised contract practice there is not the slightest reason why the stockbroker's son should waste his father's substance in paying fees to his doctor. If ever, therefore, the panel system works smoothly, the medical man will be faced with the fact that every insured person will avail himself of his privileges and obtain his treatment on contract terms. The influence of this result in limiting the income of a medical man is a very interesting matter for speculation.

We may pass over for the moment the minor troubles of the panel doctor, the absurd records which have been found impracticable, questions about certificates, and so on. But as long as the medical profession remains a weak and disunited body, medical men will have to swallow some sort of patched-up compromise on all these points. We may deal next with the distinction that has arisen between panel and non-panel practitioners.

This distinction between the two classes of practitioners was bound to arise under the Act. Instead of trying to eliminate distinctions in medical practice, it has enlarged and exaggerated the difference. The panel doctor who, it must be remembered, succumbed according to his own account unwillingly and as the result of economic pressure, is now imploring the non-panel man not to perpetuate the distinction. Surely the panel man is forgetting that it was he who made the distinction when he succumbed to circumstances, and that as he has made his bed, so must he lie on it. The distinction is unavoidable for the following reasons:—

(*a*) In former times under friendly society work the distinction existed. Every medical man avoided contract work if he could, or strove to place himself in a position in which he could dispense with it.

Under the Act there has been a great extension of contract work, and the distinction is emphasised.

(b) The limitations of panel treatment make it an inferior work. Restrictions have to be imposed on patients with regard to hours of attendance, limitations exist in drugs supplied under the panel system, and worst of all the control of a lay body for the most part ignorant of medicine deprives the panel practitioner of his former freedom.

It should be the aim of all medical men to abolish this distinction into classes. But as long as the panel exists it is impossible to do so. If the panel were abolished and the Medical Register substituted therefor it would become possible to distribute insured persons so widely amongst medical practitioners that the distinction would cease to exist. As matters are it will very likely become the desire of most panel practitioners to be freed from their service. But to very few of them will this be possible. Thence will result a deterioration in the class of men entering the profession. There will be a living for the unambitious and the mediocre in doing panel work. There will be some openings for the few who are clever enough to specialise, or are wealthy enough to confine their practice to the uninsured.

